# Production of bioactive plant secondary metabolites through in vitro technologies—status and outlook

**DOI:** 10.1007/s00253-021-11539-w

**Published:** 2021-09-01

**Authors:** Christoph Wawrosch, Sergey B. Zotchev

**Affiliations:** Department of Pharmaceutical Sciences, Division of Pharmacognosy, University of Vienna, Vienna, Austria

**Keywords:** Plant secondary metabolites, Plant tissue culture, Cell suspension, Hairy roots, Heterologous production

## Abstract

Medicinal plants have been used by mankind since ancient times, and many bioactive plant secondary metabolites are applied nowadays both directly as drugs, and as raw materials for semi-synthetic modifications. However, the structural complexity often thwarts cost-efficient chemical synthesis, and the usually low content in the native plant necessitates the processing of large amounts of field-cultivated raw material. The biotechnological manufacturing of such compounds offers a number of advantages like predictable, stable, and year-round sustainable production, scalability, and easier extraction and purification. Plant cell and tissue culture represents one possible alternative to the extraction of phytochemicals from plant material. Although a broad commercialization of such processes has not yet occurred, ongoing research indicates that plant in vitro systems such as cell suspension cultures, organ cultures, and transgenic hairy roots hold a promising potential as sources for bioactive compounds. Progress in the areas of biosynthetic pathway elucidation and genetic manipulation has expanded the possibilities to utilize plant metabolic engineering and heterologous production in microorganisms. This review aims to summarize recent advances in the in vitro production of high-value plant secondary metabolites of medicinal importance.

**Key points**

• *Bioactive plant secondary metabolites are important for current and future use in medicine*

• *In vitro production is a sustainable alternative to extraction from plants or costly chemical synthesis*

• *Current research addresses plant cell and tissue culture, metabolic engineering, and heterologous production*

**Graphical abstract:**

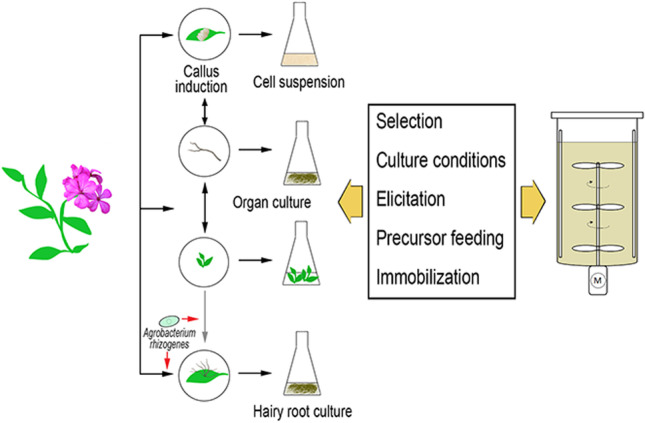

## Introduction

Mankind has been using medicinal plants since thousands of years (Fabricant and Farnsworth 2001; Kinghorn et al. 2011), and herbal medicines continue to be used around the world, particularly, but not solely, in developing countries (Robinson and Zhang 2011). Isolation of morphine from the opium poppy *Papaver somniferum* by Sertürner some 200 years ago (Klockgether-Radke 2002), and the subsequent isolation of compounds like, e.g., cocaine or digitoxine, marked the beginning of rational drug discovery (Pferschy-Wenzig and Bauer 2015), as plant-derived drugs could now be administered much more precisely compared to the crude herbal extracts (Li and Vederas 2009). Isolation and characterization of biologically active compounds from medicinal plants is ongoing, with the aim of either direct use of such plant secondary metabolites as drugs, or as templates for synthetic modification (Balunas and Kinghorn 2005; Atanasov et al. 2015; Chen et al. 2015). About 15,000 or ca. 5% of all estimated species of higher plants have documented medicinal use (Cordell and Colvard 2005). This knowledge, together with the potential use of yet unexplored species are of great value for the discovery of new bioactive natural products (Fabricant and Farnsworth 2001; Rates 2001; Cordell and Colvard 2005; Albuquerque et al. 2012). The plant microbiome has been recognized as a source of novel bioactive compounds, too (Atanasov et al. 2021; Oberhofer et al. 2021; Bekiesch et al. 2021).

**Fig. 1:**
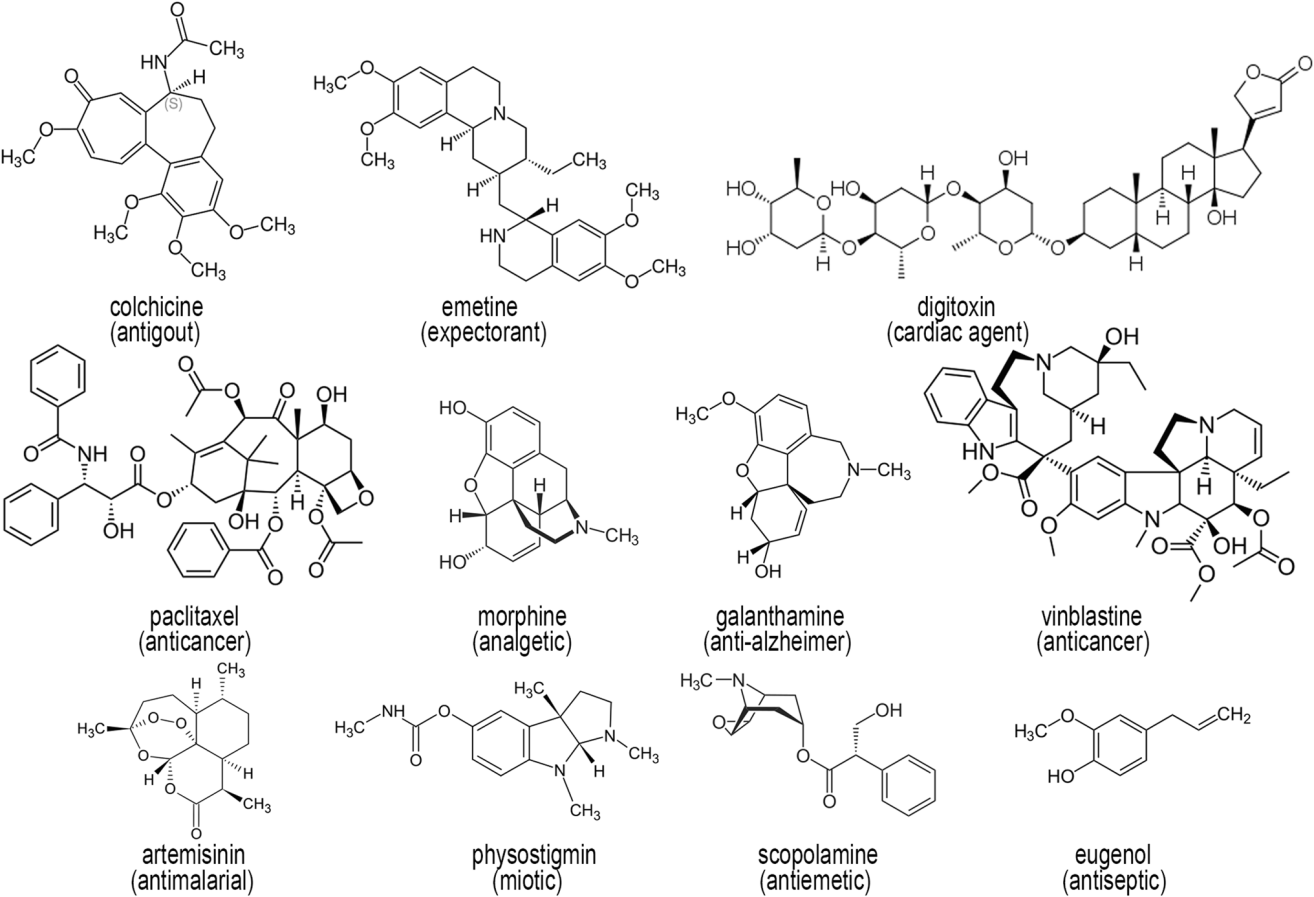
Chemical structures of some bioactive plant secondary metabolites used in medicine

Some of the plant secondary metabolites extensively used today in medical practice are shown in Fig. 1. In view of rising extinction rates of plant species (Brower 2008; Urban 2015) and of the importance of biodiversity conservation (Kingston 2011), issues of supply of plant material, and, consequently, plant-derived compounds, must be considered as early as possible (McChesney et al. 2007; Salim et al. 2008). Because of the frequently found structural complexity of plant secondary metabolites, very often their chemical synthesis is economically unfeasible (Staniek et al. 2014; Dziggel et al. 2017). In most cases, only low to minimal amounts of the natural products of interest occur in the respective species, and due to various plant-specific and environmental factors the content in wild-growing plants can be highly variable (Canter et al. 2005; Pferschy-Wenzig and Bauer 2015; Ochoa-Villarreal et al. 2016). Therefore, alternatives to harvesting the plant material from natural resources are highly desired. The application of plant tissue culture techniques offers a range of opportunities for a sustainable access to natural products.

A number of recent reviews deals with plant tissue culture for the production of secondary compounds, for example by Chandran et al. (2020), Gutierrez-Valdes et al. (2020), Marchev et al. (2020), Cardoso et al. (2019), Kreis (2019), Rahmat and Kang (2019), and more. The present mini-review on the one hand highlights recent achievements in the production of medically important plant secondary metabolites using various in vitro technologies, including most recent approaches like metabolic engineering and heterologous production in microorganisms. In addition, progress in the optimization of in vitro productivity will be analyzed on the basis of selected compounds.

## Production via plant in vitro cultures

**Fig. 2:**
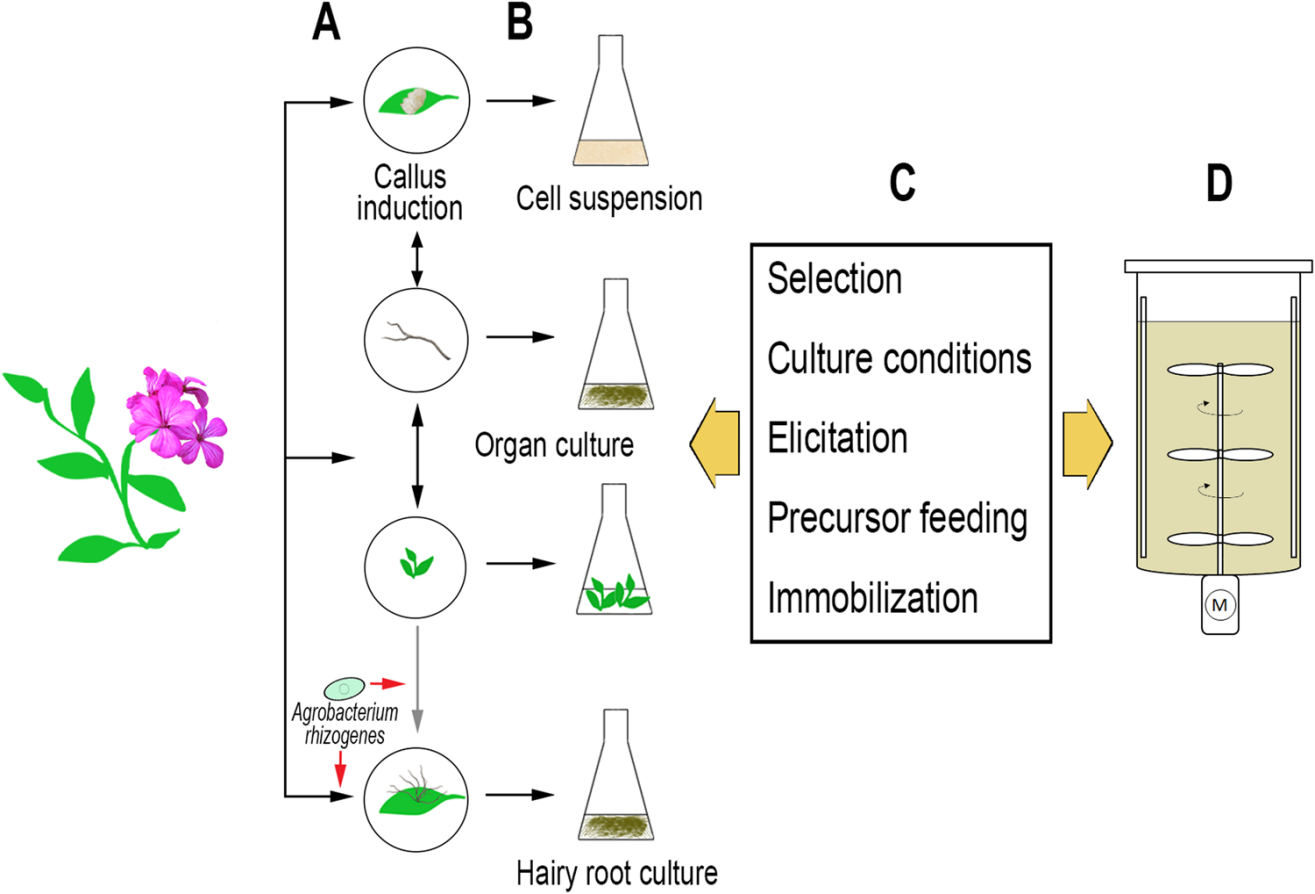
Methods for the production of bioactive secondary metabolites through plant in vitro culture. **A** Establishment of in vitro cultures after surface sterilization of plant material. Callus formation can also be induced on material from organ cultures. Reversely, organs like roots or shoots can be regenerated from callus. Hairy roots are obtained by infection of sterilized donor plant, or in vitro cultivated material, with Agrobacterium rhizogenes. **B** Multiplication of primary callus/organs/roots, first selection, and establishment of liquid cultures. **C** Selection of high yielding lines and optimization of culture conditions (nutrient medium composition, inoculum density, temperature, light, agitation and aeration). Strategies like elicitation, precursor feeding, or immobilization are pursued to further improve productivity. **D** The bioreactor design will depend on the culture type: stirred tanks, but also airlift and bubble column reactors for cell suspensions; mist or spray reactors and temporary immersion systems for organ cultures (including hairy roots)

The methodology for initiating the in vitro cultures of plant cells, tissues, and organs is nowadays well established, and a brief overview of the most important procedures is illustrated in Fig. 2. The cultures can be initiated from parts of the whole plant, or from seeds which are germinated aseptically. A prerequisite for introducing any plant material into in vitro culture is the surface sterilization in order to eliminate adhering microorganisms, this is usually performed with aqueous solutions of disinfectants like, e.g., sodium hypochlorite. Suitable explants are then inoculated on a semi-solid medium containing adequate amounts of nutrients and, most importantly, plant growth regulators, and cultivated under controlled environmental conditions. Depending on the starting material and the nutrient medium, different types of cultures can be established which are classified as cell cultures (cell suspensions, protoplasts, or gametic cells), tissue cultures (callus or differentiated tissues), or organ cultures (e.g., shoots, roots, or zygotic embryos). On the one hand, the formation of callus (dedifferentiated cell masses) can be induced, and upon transfer to liquid medium clumps of callus might disintegrate into small aggregates and single cells, whereby cell suspension cultures are obtained. As callus typically is quite heterogeneous with respect to the biochemical properties of the cells, suspension cultures should be started from small callus aggregates, so that homogeneous cell lines, desirably with a fast growth, can evolve. Cell suspension cultures are a potential source for the production of high-value plant secondary metabolites, and during the past decades cultures from many medicinal plants have been established (see, e.g., Karuppusamy 2009). On the other hand, shoot tips or axillary buds of a donor plant will grow to shoot cultures on an appropriate nutrient medium. When such shoots are regularly dissected and bud-bearing segments subcultivated, it is possible to achieve high multiplication rates, and the micropropagation especially of ornamental plants is nowadays applied widely on a commercial basis (Preil 2003). For the purpose of secondary metabolite production, most often research focuses on cell suspension cultures because of comparatively fast growth and the possibility of scale-up to bioreactor systems. However, it must be considered that in higher plants some biochemical traits may only be fully developed in specific organs, or during particular stages of development. Thus, the accumulation of such metabolites may depend on the presence of certain cell types or organelles, and the expression and regulation of biosynthetic genes (Kreis 2007). For this reason, past and ongoing studies also deal with the application of in vitro cultured organs like shoots, roots, or embryos for the production of plant metabolites, although the high costs of large-scale cultivation because of the need for special bioreactors have hitherto largely impeded a commercial implementation (Verpoorte et al. 2002).

Hairy roots cultures are of particular interest for the production of compounds which are biosynthesized in roots. The hairy roots disease is caused by the soil-borne *Agrobacterium rhizogenes* which upon infection transfers genes into the host plant, some of which encode for growth hormone biosynthesis. This procedure can be carried out in the lab, and the resulting hairy root cultures feature rapid growth in simple media, genetic stability, and the capacity of secondary metabolite production in equal or higher amounts than the host plant (Kumar 2015). In addition, by manipulating the bacterial Ri plasmid genes of interest can be introduced into hairy roots. Over the past few decades, hairy roots have gained increased interest as a biotechnological tool not only for the production of plant secondary metabolites, but also for the elucidation of biosynthetic pathways, studies of rhizosphere physiology and biochemistry, metabolic engineering, and phytoremediation (Talano et al. 2012; Gutierrez-Valdes et al. 2020). In vitro they exhibit fast growth, are genetically stable, and can accumulate the same secondary metabolites as the parent plant. Hairy roots of over 100 medicinal plants have been investigated so far (see for example (Dhiman et al. 2018), and a few of the most recent studies are reviewed below.

Most often, in a given plant in vitro culture the content of a desired secondary metabolite can initially be quite low, particularly in callus and cell suspension cultures. Hence, when cell cultures are to be developed, screening for high yielding and fast-growing lines should be undertaken—this also applies to hairy roots because an infection experiment with *A. rhizogenes* usually results in the emergence of several distinct hairy root lines which may vary in properties like growth and secondary metabolite accumulation. Next, for any type of in vitro cultures the optimization of the culture conditions with respect to factors such as nitrogen source, carbon source, plant growth regulators, medium pH, temperature, light and oxygen is likely to improve product formation. Elicitation is an important and expedient strategy to enhance the productivity of the culture. Elicitors are compounds that stimulate various mechanisms of plant defense and thus promote the biosynthesis of secondary metabolites to protect the cell and the whole plant (Ramirez-Estrada et al. 2016). Biotic elicitors are substances of biological origin, like polysaccharides originated from plant cell walls and micro-organisms, fungal and bacterial lysates, or yeast extract. Abiotic elicitors are of non-biological origin and include physical (osmotic stress, UV light, ultrasound, etc.), chemical (e.g., heavy metals or mineral salts) and hormonal factors like salicylic acid or jasmonates.

The in vitro production of secondary metabolites has a number of distinct advantages over the extraction from whole plants. There are no seasonal constraints and a predictable, reliable, and continuous year-round production is possible. It is of special interest when a plant species is slow growing or difficult to cultivate at all, and when the content of the desired compound in planta is exceedingly low. In some cases, it has been shown that upon optimization product accumulation in vitro can exceed the content in whole plants. However, in view of the fact that many authors claim that their in vitro system will allow for the commercial production of the respective compound, a few economic considerations are necessary. As recently discussed by Lange (2018), a number of factors are likely to affect how a given target compound is obtained on an industrial scale. Although many natural compounds have a very complex chemical structure, just as many exhibit a comparatively uncomplicated structure. In the latter case, chemical synthesis of the compound will be economically superior to a plant-based production (both through extraction from plant raw materials, or through plant in vitro culture). Concerning the isolation of compounds from plant material, criteria like price of the raw material, concentration of the desired natural product, and costs of the extraction/purification process are further factors to consider, as are market size and regulatory provisions.

Recent achievements in the production of bioactive secondary metabolites in cell suspension cultures, as well as organ cultures (including hairy roots), are reviewed in the following sections and summarized in Table 1.

**Table 1:** Bioactive secondary metabolites obtained via plant in vitro cultures

Compound	Plant species	Product yield	Culture type	Reference
Alkaloids				
Berberine	*Argemone mexicana*	ca. 1.3 mg^.^g^−1^ DW	SH	Monforte-González et al. (2019)
Camptothecin	*Ophiorrhiza mungos*	0.86 mg⋅g^−1^ DW 1.12 mg^.^g^−1^ DW	AR S	Deepthi and Satheeshkumar (2017a); Deepthi and Satheeshkumar (2017b)
Capsaicin & dihydrocapsaicin	*Capsicum chinense*	2.87 / 1.03 mg^.^g^−1^ DW	S	Kabita et al. (2020)
Indole alkaloids	*Isatis tinctoria*	3.15 mg^.^g^−1^ DW	HR	Gai et al. (2019)
Phenylpropanoids				
Chlorogenic acid derivatives	*Gardenia jasminoides*	20.98 mg^.^g^−1^ DW	S	Liu et al. (2018)
Cichoric acid	*Ocimum basilicum*	6.90 mg^.^g^−1^ DW	S	Açıkgöz (2020)
Flavonoids (total)				
	*Ficus deltoidea* var. *kunstleri*	3.3 mg^.^g^−1^ DW	S	Haida et al. (2019)
Flavonoids (chrysin, wogonin and baicalein)	*Scutellaria bornmuelleri*	163.42 mg^.^g^−1^ DW	HR	Gharari et al. (2020a)
Flavonoids, phenolics, polysaccharides	*Oplopanax elatus*	53.87 / 30.10 / 192.64 mg^.^g^−1^ DW	AR	Jiang et al. (2017)
Isoflavones	*Trifolium pratense*	23.53 mg^.^g^−1^ DW	IVP	Reis et al. (2018)
Isoquercetin	*Ocimum basilicum*	3.72 mg^.^g^−1^ DW	S	Açıkgöz (2020)
Rosmarinic acid	*Ocimum basilicum*	22.53 mg^.^g^−1^ DW	S	Açıkgöz (2020)
	*Salvia leriifolia*	6.41 mg^.^g^−1^ DW	S	Modarres et al. (2018)
Rutin	*Ocimum basilicum*	6.54 mg^.^g^−1^ DW	S	Açıkgöz (2020)
Lignans				
Lignans (total)	*Linum album*	122.73 µ^.^g^−1^ FW	HR	Ahmadian Chashmi et al. (2016)
Dehydrodiconiferyl alcohol glucoside	*Linum usitatissimum*	21.6 mg^.^g^−1^ DW	AR	Anjum et al. (2020)
Guaiacylglycerol-ß-coniferyl alcohol ether glucoside	*Linum usitatissimum*	4.9 mg^.^g^−1^ DW	AR	Anjum et al. (2020)
Lariciresinol diglucoside	*Linum usitatissimum*	11.9 mg^.^g^−1^ DW	AR	Anjum et al. (2020)
Podophyllotoxin / 6-methoxypodo-phyllotoxin	*Linum album*	47 / 9.5 µg^.^g^−1^ DW 135 µg^.^g^−1^ DW / 15 mg^.^g^−1^ DW ca. 60 µg^.^g^−1^ DW / 9.5 mg^.^g^−1^ DW	S AR HR	Lalaleo et al. (2018) Lalaleo et al. (2018) Lalaleo et al. (2018)
Secoisolariciresinol diglucoside	*Linum usitatissimum*	5.5 mg^.^g^−1^ DW	AR	Anjum et al. (2020)
Stilbenes				
Mulberroside A	*Morus alba*	31.59 mg^.^L^−1^	S	Komaikul et al. (2019)
Terpenoids				
Artemisinin	*Artemisia annua*	10.86 mg^.^g^−1^ DW 9.33 mg^.^L^−1^	S S	Zebarjadi et al. (2018) Salehi et al. (2019a)
		1.12 mg^.^g^−1^ DW	HR	Patra and Srivastava (2016)
Betulinic acid	*Lantana camara*	0.117 mg^.^g^−1^ DW	S	Kumar et al. (2016)
Bilobalide	*Ginkgo biloba*	78 mg^.^g^−1^ DW	S	Sukito and Tachibana (2016)
Ginkgolide A / B / C	*Ginkgo biloba*	79 / 71 / 7.5 mg^.^g^−1^ DW	S	Sukito and Tachibana (2016)
Ginsenosides	*Panax ginseng*	2.62–9.04 mg^.^g^−1^ DW 32.46 mg^.^g^−1^ DW	S AR	Le et al. (2019) Murthy et al. (2018)
	*Panax quinquefolius*	87.6 mg^.^L^−1^	S	Biswas et al. (2016)
	*Panax sikkimensis*	222.2 mg^.^L^−1^	S	Biswas et al. (2018)
Oleanolic acid	*Lantana camara*	1.43 mg^.^g^−1^ DW	S	Kumar et al. (2016)
	*Salvia fruticosa*	5.76 mg^.^g^−1^ DW	S	Kümmritz et al. (2016)
Oleanolic acid glycosides	*Calendula officinalis*	52.52 mg^.^g^−1^ DW	HR	Alsoufi et al. (2019)
Paclitaxel	*Corylus avellana*	404.5 µg^.^L^−1^ 402.4 µg^.^L^−1^ 3.2 µg^.^g^−1^ DW	S S HR	Salehi et al. (2019b) Farhadi et al. (2020) Jalalipour Parizi et al. (2020)
	*Taxus x media*	2.47 mg^.^g^−1^ DW	HR	Sykłowska-Baranek et al. (2019)
Steviol glycosides (stevioside + rebaudioside A)	*Stevia rebaudiana*	92.58 mg^.^g^−1^ DW	AR	Ahmad et al. (2018)
Ursolic acid	*Lantana camara*	3.87 mg^.^g^−1^ DW	S	Kumar et al. (2016)
	*Salvia fruticosa*	10.77 mg^.^g^−1^ DW	S	Kümmritz et al. (2016)
Withanolides	*Withania somnifera*	14.2 mg^.^g^−1^ DW	S	Ahlawat et al. (2017)
Other				
L-Dopa	*Hybanthus enneaspermus*	12.64 mg^.^g^−1^ DW	AR	Sathish et al. (2020)

*AR* adventitious root culture, *DW* dry weight, *HR* hairy roots, *IVP* in vitro cultivated plant, *S* cell suspension culture, *SH* shoot culture

## Alkaloids

Among the large variety of secondary metabolites synthesized by plants, alkaloids exhibiting various pronounced biological activities are being extensively used in medicine. Most prominent examples are atropine and scopolamine, quinine, ephedrine, or the opium poppy alkaloids codeine and morphine, only to name a few. Despite the importance of this class of compounds, only limited studies on their in vitro production have been reported. Berberine is a benzylisoquinoline alkaloid found in many plant genera like, e.g., *Berberis, Argemone, Eschscholzia*, or *Coptis* (Neag et al. 2018), and exhibiting effects on the cardiovascular, gastrointestinal, endocrine, immune, and central nervous systems (Imanshahidi and Hosseinzadeh 2008). In a recent study on berberine formation in the in vitro cultures of prickly poppy (*Argemone mexicana* L., *Papaveraceae*) the authors demonstrated that alkaloid biosynthesis is related to the level of tissue organization (Monforte-González et al. 2019). Berberine was found in leaves and stems used as initial explants, and in shoots and roots regenerated from callus (1.3 mg⋅g^−1^ DW and ca. 0.8 mg⋅g^−1^ DW, respectively). Research in the field of the in vitro production of berberine is carried out since some decades. Notably, selected cell lines of *Coptis japonica* with a production of up to 13.2% (DW) have been reported by Sato and Yamada (1984).

The monoterpenoid pentacyclic alkaloid camptothecin is a cytotoxic compound originally isolated from the Chinese tree *Camptotheca acuminata* (Liu et al. 2015) but also found in other plant species. While camptothecin itself is not used as a drug, it serves as starting material for the semisynthetic preparation of important antitumor drugs like, e.g., topotecan and irinotecan (Kacprzak 2013). Deepthi and Satheeshkumar (2017b) have investigated camptothecin formation in suspension cultures of *Ophiorrhiza mungos*. Through cell line selection, optimization of the nutrient medium, and elicitation with jasmonic acid, the authors achieved a camptothecin production of 1.12 mg⋅g^−1^ DW (dry weight) compared to 0.06 mg⋅g^−1^ DW in the original cell line. The accumulation of camptothecin in adventitious roots of *Ophiorrhiza mungos* has been studied by Deepthi and Satheeshkumar (2017a), who showed that camptothecin production was influenced by the nutrient medium (concentration of major nutrients, plant growth regulators) and by inoculum size. Under optimized conditions the in vitro cultures produced 0.86 mg⋅g^−1^ DW of the alkaloid, which was well above the concentration of 0.15 mg⋅g^−1^ DW found in the roots of the donor plant used for culture establishment. Studies on the in vitro production of camptothecin started more than 40 years ago (for a review see Kai et al. 2015). In cell suspension cultures of *C. acuminata* treated with a protein elicitor from the fungus *Phytophthora boehmeriae*, Lu et al. (2011) achieved the formation of 12 mg⋅g^−1^ DW camptothecin. Viewed in this light, the recent findings on camptothecin in vitro production cannot be regarded as a progress towards a possible large-scale production of the compound through plant tissue culture.

The root of *Isatis tinctoria* (synonym *I. indigotica*) has been used since centuries in Traditional Chinese Medicine for the treatment of influenza and hepatitis. It has also shown significant activity in the treatment of SARS (Xiao et al. 2015). The numerous secondary metabolites identified in the plant include various alkaloids and flavonoids (Hamburger 2002). In a hairy root line of *I. tinctoria* Gai et al. (2019) recently obtained 3.15 mg.g^−1^ DW alkaloids (epigoitrin, isatin, indole-3-carboxaldehyde, tryptanthrin, indigo, and indirubin) after elicitation with 142.61 µM salicylic acid.

Capsaicin and several related compounds are the pungent principles of chili peppers (species from the genus *Capsicum*). Beside the use of chili as a spice, capsaicin has analgesic properties and possesses beneficial effects in the treatment of a number of diseases (Fattori et al. 2016). A recent study by Kabita and co-authors (Kabita et al. 2020) deals with capsaicinoid biosynthesis in cell suspension cultures of *Capsicum chinense* Jacq. cv. ‘Umorok’. Elicitation of the cells with chitosan resulted in levels of 2.87 mg^.^g^−1^ FW (fresh weight) capsaicin and 1.03 mg^.^g^−1^ FW dihydrocapsaicin. Although biotechnological research on in vitro production of capsaicin is now going on since decades (see, e.g., review by Kehie et al. 2015), this metabolite cannot be regarded as a target compound for the in vitro production. Depending on cultivar and processing of the fruits, capsaicin contents from 0.5 to more than 4% DW have been reported (Yaldiz et al. 2010), hence it seems a debatable point whether biotechnological capsaicin production can compete with the extraction from chili peppers.

## Flavonoids and phenolic acids

Flavonoids represent another large group of natural products which are found in all higher plants. These phenolic compounds exhibit a multitude of biological activities and are used in nutrition as well as for pharmaceutical applications (Sticher 2007a; Panche et al. 2016). Many plants also produce phenolic acids with various activities like, e.g., anti-inflammatory, anti-oxidative, and antiviral (Sticher 2007a). In this section, recent studies on the formation of flavonoids and phenolic acids in plant tissue cultures are presented, followed by some critical comments.

Haida et al. (2019) investigated the effects of initial inoculum size, cell aggregation and pH of the nutrient medium on the total flavonoid content in a cell suspension culture of *Ficus deltoidea* var. *kunstleri*. The authors reported the highest content of 3.3 mg^.^g^−1^ DW in cell suspension fractions with aggregates sized 500–750 µm and thus confirmed that although fine cell suspensions are usually preferred, aggregation can sometimes improve product formation (Mustafa et al. 2011). Chlorogenic acid derivatives are widely distributed phenolic plant compounds with antioxidant, anti-inflammatory, antiallergic and hydrocholeretic properties (Sticher 2007a; Upadhyay and Rao 2013). Liu et al. (2018) studied the effects of elicitation with salicylic acid and methyl jasmonate on the formation of six chlorogenic acid derivatives in suspension cultures of *Gardenia jasminoides*. Treatment with 200 µM methyl jasmonate resulted in the accumulation of nearly 21 mg^.^g^−1^ DW of the compounds, which was about twofold the yield of the control. Sweet basil (*Ocimum basilicum* L.) is a well-known herb widely used for culinary purposes and also as a herbal remedy for treating headaches, coughs, digestive, and nervous disorders (Majdi et al. 2020). The formation of flavonoids and phenolic acids in *O. basilicum* cell suspension cultures as influenced by elicitors (AgNO_3_, CdCl_2_, and yeast extract) was studied by Açıkgöz (2020). Depending on the treatment, the author reported an enhanced flavonoid formation of up to 6.54 mg^.^g^−1^ DW rutin and 3.72 mg^.^g^−1^ DW isoquercetin. The accumulation of rosmarinic acid (22.53 mg^.^g^−1^ DW) and cichoric acid (6.90 mg^.^g^−1^ DW) was stimulated through elicitor treatment, too. However, no figures are given of the content of these compounds in planta. This hampers a comparative evaluation of the results, especially because of the multitude of basil varieties and cultivars which can vary considerably in their content of bioactive compounds (Makri and Kintzios 2008; Srivastava et al. 2014). Modarres et al. (2018) reported the formation of 6.41 mg^.^g^−1^ DW rosmarinic acid in cell suspension cultures of *Salvia leriifolia*. Elicitation of a *Isatis tinctoria* hairy root line (“ITHRL V”) with 179.54 µM methyl jasmonate resulted in the accumulation of 4.96 mg^.^g^−1^ DW flavonoids (rutin, neohesperidin, buddleoside, liquiritigenin, quercetin, isorhamnetin, kaempferol, and isoliquiritigenin)(Gai et al. 2019). The formation of isoflavones in the in vitro cultivated whole plants of *Trifolium pratense* was investigated by Reis et al. (2018). After a cultivation period of 60 days in vitro plants showed a higher total isoflavone content (23.55 mg^.^g^−1^ DW) than the two analysed wild plants (14.52 and 16.51 mg^.^g^−1^ DW, respectively). Interestingly, only a low amount of the compound daidzein, which was found in the in vitro plants at a level of 17.59 mg^.^g^−1^ DW, could be detected in one of the wild plant samples (0.37 mg^.^g^−1^ DW). The authors concluded that in vitro plants could be a source for the commercial production of red clover isoflavones. However, a comparison of the in vitro isoflavone content to only two wild plant samples cannot be regarded as statistically meaningful. Also, it has been reported earlier that the isoflavone content in different plant parts of *T. pratense* varied according to the vegetation period (Gikas et al. 2008). Last but not least, red clover is one of the most important forage plants which is cultivated worldwide (Boller et al. 2010), and the isoflavone content can be as high as 2.5% DW (Saloniemi et al. 1995). Hence, it seems unprobable that in vitro production of these isoflavones could compete to extraction from plant material.

The roots of *Oplopanax elatus*, a member of the *Araliaceae* family, are used medicinally in China, Korea, and Russia for the treatment of various disorders like, e.g., chronic fatigue syndrome, cardiovascular diseases, diabetes mellitus and rheumatism. Among various compounds such as essential oils, saponins, or anthraquinones, the roots also contain 0.9% flavonoids (Shikov et al. 2014). The formation of flavonoids, phenolics and polysaccharides in adventitious root cultures of *O. elatus* was investigated by Jiang et al. (2017). Upon elicitation with 200 µM methyl jasmonate they observed the accumulation of 53.87 mg^.^g^−1^ DW flavonoids and 192.64 mg^.^g^−1^ DW polysaccharides, while treatment with 100 µM salicylic acid led to the production of 30.10 mg^.^g^−1^ DW phenolics. The authors expect this adventitious root system to be used commercially, but it is not known whether only the three compound classes measured in this study are responsible for the biological activities. Hence, while a flavonoid content of 5% as determined in the adventitious roots is undoubtedly high, further studies are necessary. Species of the genus *Scutellaria* (*Lamiaceae*) are widely used medicinally in East Asia due to is anti-inflammatory, antithrombotic, sedative, antiviral, and antioxidant effects (Shang et al. 2010). A recent paper deals with the effects of different elicitors (methyl jasmonate, methyl-ß-cyclodextrin, and chitosan) on the production of the three main flavonoids chrysin, wogonin, and baicalein in hairy roots of *Scutellaria bornmuelleri* (Gharari et al. 2020a). The best results were obtained upon treatment with 50 mg^.^L^−1^ chitosan together with 100 µM methyl jasmonate, which yielded a total of 163.42 mg^.^g^−1^ DW flavonoids (56.47 mg^.^g^−1^ DW chrysin + 27.26 mg^.^g^−1^ DW wogonin + 79.69 mg^.^g^−1^ DW baicalein). Hence, the flavonoid yield of this hairy roots system was clearly superior to intact roots which were shown to contain a total of only 0.0328 mg^.^g^−1^ DW flavonoids (Gharari et al. 2020b).

While apparently much current research is focused on the production of flavonoids through plant tissue culture, it is questionable whether these secondary metabolites are suitable target compounds. Firstly, flavonoids are found in often considerable concentrations in a large number of plants (see, e.g., Panche et al. 2016). Indeed, some compounds are extracted commercially from plant raw materials, like, e.g., hesperidin from citrus peel (Cheigh et al. 2012), or rutin from buckwheat or *Eucalyptus* sp. (e.g., Shafi and Ikram 1982). Herein, the development of improved extraction processes (Chávez-González et al. 2020) will further improve the economic efficiency of flavonoid production from plant materials. Also, considerable progress has been achieved in the heterologous production of flavonoids in microbial hosts (see for example Trantas et al. 2015), and some examples will be presented below.

Similarly, quite a number of studies deal with the production of rosmarinic acid and other phenolic acids. Research into the in vitro formation of rosmarinic acid has begun many decades ago. As reviewed by Pezeshki and Petersen (2018), the first respective studies with callus and cell suspension cultures of *Coleus blumei* were published in 1977, and in 1985 a bioreactor process with a yield of 21% DW or 5.5 g^.^L^−1^ was described (Ulbrich et al. 1985). Other production processes with various plant genera have since been developed, like, e.g., cell suspension cultures of *Salvia officinalis* with a productivity of 6.4 g^.^L^−1^ rosmarinic acid (Hippolyte et al. 1992). It has been considered that none of these highly productive systems have been commercialized because of medicinal applications of the pure substance have not been developed (Pezeshki and Petersen 2018). Nevertheless, although recently reported in vitro systems quoted above cannot compete with the early processes based on *Coleus* or *Salvia*, the authors still claim that their findings could have commercial potential.

## Stilbenes

Mulberroside A (the diglucoside of oxyresveratrol) is a stilbene representing one of the main bioactive compounds in mulberry (*Morus alba* L.). Numerous potential health benefits are attributed to this group of natural products known as phytostilbenes (Reinisalo et al. 2015). Komaikul et al. (2019) investigated phytostilbene formation in cell suspension cultures of mulberry cultivated in different bioreactors. A level of 31.59 mg^.^L^−1^ mulberroside A was achieved, and the authors demonstrated that the production was influenced by factors like aeration, biomass circulation in the bioreactor, and endogenous enzymatic hydrolysis (which can occur upon cell disruption). Mulberry contains up to 1.3% mulberroside A (Jiang et al. 2018) and the plant is fast-growing and widely distributed in the Northern and Southern hemispheres (Wen et al. 2019). Hence, it is questionable whether a biotechnological production of mulberroside A can be economically competitive to the extraction from raw plant material.

## Lignans

Among the various phenylpropanoids which occur in the plant kingdom, lignans are structurally diverse compounds which exhibit potent biological activities (Barker 2019). The clinically important anticancer drugs etoposide, etopophos and teniposide have been developed from podophyllotoxin, a lignan found in species of the genus *Podophyllum* (Canel et al. 2000). The species *Linum album* contains the lignans podophyttotoxin (PTOX) and 6-methoxypodophyllotoxin (6-MPTOX) (Schmidt et al. 2010). Lalaleo et al. (2018) investigated lignan formation in different types of *L. album* in vitro culture. They reported the accumulation of 47 µg^.^g^−1^ DW PTOX and 9.5 µg^.^g^−1^ DW 6-MPTOX in cell suspension cultures, while adventitious root cultures were shown to accumulate the lignans podophyllotoxin and 6-methoxypodophyllotoxin at levels of 135 µ^.^g^−1^ DW and 15 mg^.^g^−1^ DW, respectively. Anjum et al. (2020) studied the formation of lignans and neolignans in adventitious root cultures of flax. They could detect lignans at levels of 11.9 mg^.^g^−1^ DW (lariciresinol diglucoside) and 5.50 mg^.^g^−1^ DW (secoisolariciresinol diglucoside), and neolignans at concentrations of 21.6 mg^.^g^−1^ DW (dehydrodiconiferyl alcohol glucoside) and 4.9 mg^.^g^−1^ DW (Guaiacylglycerol-ß-coniferyl alcohol ether glucoside). The formation of lignans was also investigated in hairy root cultures of *Linum album*. Lalaleo et al. (2018) reported the accumulation of nearly 60 µg^.^g^−1^ DW podophyllotoxin and 9.5 mg^.^g^−1^ DW 6-methoxypodophyllotoxin, these were about half the levels of those found in adventitious roots of *L. album*, as mentioned earlier. Ahmadian Chashmi and co-authors (2016) studied the formation of lignans in feeding experiments with hairy root cultures of *L. album*. After addition of 2 mM coniferylaldehyde they detected 107.61 µg^.^g^−1^ FW lariciresinol, 8.7 µg^.^g^−1^ FW pinoresinol, and 6.42 µg^.^g^−1^ FW podophyllotoxin. The alpine plant Edelweiss (*Leontopodium nivale* ssp. *alpinum*) contains, amongs other compounds, the bioactive lignan leoligin at concentrations of 0.05–0.1 mg^.^g^−1^ DW (Wawrosch et al. 2013). Elicitation of a hairy root line of Edelweiss with 6% sucrose resulted in accumulation of 0.678 mg^.^g^−1^ DW leoligin (Wawrosch et al. 2014).

Research on the in vitro production of lignans, especially of podophyllotoxin as important starting product for the semi-synthetic manufacture of anti-cancer drugs, stretches back several decades (Ionkova 2007). Notably, cell suspension cultures of *Linum album* were shown to accumulate up to 0.5% lignans, with podophyllotoxin as major compound (Smollny et al. 1998). A cell culture of *Linum nodiflorum* even accumulated 1.7% DW of 6-methoxypodophyllotoxin (Kuhlmann et al. 2002). In view of these early reports, the recent findings quoted above do not represent a progress towards significantly increased lignan production in plant tissue cultures.

## Terpenoids

Isoprenoids or terpenoids are ubiquitous natural compounds found in most organisms, with the majority occurring in plants. Terpenoids show manifold biological activities and are used pharmaceutically and as additives to food and cosmetic products (Sticher 2007b; Tetali 2019). The sesquiterpene lactone, artemisinin, is an important antimalaria drug occurring at low levels in sweet wormwood, *Artemisia annua* L. Due to the high demand for this compound and the complex total chemical synthesis, alternative sources including in vitro cultures of *A. annua* are being sought (Ali et al. 2017). Recently, Zebarjadi et al. (2018) could enhance the formation of artemisinin in cell suspension cultures of *A. annua* to 10.86 mg^.^g^−1^ DW through elicitation with abscisic acid. Similarly, Salehi and co-authors (2019a) reported a production of 9.33 mg^.^L^−1^ upon elicitation with coronatine and sorbitol. However, in both cases the previously reported yield of 110.2 mg^.^L^−1^ (Baldi and Dixit 2008) could not be surpassed. The high productivity reported by Baldi and Dixit (2008) was based on a biomass production of 15.2 g^.^L^−1^ which was approx. fourfold higher than the one achieved by Salehi et al. (2019a). In addition, Baldi and Dixit (2008) combined elicitation (methyl jasmonate) with precursor feeding (mevalonic acid lactone). Patra and Srivastava (2016) investigated artemisinin formation in *A. annua* hairy roots cultivated in bioreactors. Through a combination of submerged growth in liquid medium for 5 days, followed by 15 days in a nutrient mist reactor, the obtained a yield of 1.12 mg^.^g^−1^ DW, equivalent to 25.78 mg^.^L^−1^ artemisinin. Due to the importance of this antimalaria drug, research into efficient production methods of artemisinin is promoted since decades (see, e.g., reviews by Wani et al. 2021; Badshah et al. 2018; Ikram and Simonsen 2017). While much higher production rates than in plant cell and organ cultures have been achieved in microbial systems (see below), the biotechnological production of artemisinin cannot yet compete with the large-scale extraction from *A. annua* plant material.

Paclitaxel (Taxol®) is an indispensable anti-cancer drug originally prepared from the bark of the Pacific yew (*Taxus brevifolia* Nutt). Due to the low content in the natural source, over the past decades alternative sources for this diterpenoid compound were developed, and since several years cell suspension cultures of *Taxus* species are used for the industrial production of paclitaxel and related compounds (McElroy and Jennewein 2018). Attempts to increase the in vitro yields of paclitaxel are still ongoing. Sykłowska-Baranek et al. (2019) investigated paclitaxel formation in two hairy root lines of *Taxus* x *media*, one of which harbored a gene for taxadiene synthase (*TXS*). In their study they evaluated the effects of various elicitor treatments and a perfluorodecalin-supported two liquid-phase culture system on product formation and expression pattern of three paclitaxel biosynthetic genes. The authors reported a yield of 2.47 mg^.^g^−1^ DW paclitaxel in the *TXS*-harboring hairy root line upon elicitation with 100 µM methyl jasmonate, 10 µM sodium nitroprusside, 100 µM L-phenylalanine, and 30 g^.^L^−1^ sucrose, with a degassed perfluorodecalin phase. By comparison, 0.378 mg^.^g^−1^ DW paclitaxel and a total taxane content of 1.59 mg^.^g^−1^ DW were detected in the needles of one out of 17 screened cultivars of *Taxus* x *media* (Wang et al. 2006). Through co-culture of *Corylus avellana* (hazelnut) cells with the endophytic paclitaxel-producing fungus *Epicoccum nigrum* strain YEF_2_ Salehi and co-authors (Salehi et al. 2019b) achieved the production of 404.5 µg^.^L^−1^ paclitaxel, which was significantly more than in the control monocultures. Farhadi et al. (2020) demonstrated that elicitation of a hazelnut cell suspension culture with a cell wall preparation of *Coniothyrium palmarum*, together with the application of methyl-ß-cyclodextrin to enhance product secretion into the culture medium, led to the formation of 402.4 µg^.^L^−1^ paclitaxel. This was a nearly sixfold increase when compared to the untreated control. Hairy root cultures of *Corylus avellana* have been studied by Jalalipour Parizi et al. (2020). They evaluated three *A*. *rhizogenes* strains and six culture media and obtained a maximum yield of 3.2 µg^.^g^−1^ DW paclitaxel. The current knowledge about paclitaxel, including its biotechnological production, has recently been reviewed by McElroy and Jennewein (2018). Nearly two decades ago suspension cultures of *Taxus chinensis* were reported to yield 565 mg^.^L^−1^ (29.3 mg^.^g^−1^ DW) paclitaxel upon repeated elicitation with methyl jasmonate in a bioreactor (Wang and Zhong 2002). Hence, the productivity of the in vitro systems reported in the recent studies quoted above was far from the yields achieved in 2002.

Ginseng (*Panax ginseng* C.A. Meyer and other *Panax* species) is an important medicinal plant widely used in Asian countries since a long time, with increasing popularity in many other countries. Because of the time-consuming and laborious field cultivation biotechnological strategies for the production of ginsenosides, the main ginseng compounds, have been investigated for a considerable time (Wu and Zhong 1999; Murthy et al. 2014). Since 1988 ginseng cells are cultivated on an industrial scale in Japan for the production of ginsenosides (Hibino and Ushiyama 1999). Le et al. (2019) investigated ginsenoside formation in long-term (20 years) and short-term (1 year) suspension cultures of *P. ginseng* and compared the accumulation profiles of the main compounds of the protopanaxadiol and protopanaxatriol types. At different time points of the cultivation period they found total ginsenoside contents to range from 4.70 to 8.00 mg^.^g^−1^ DW in short-term cultures and from 2.62 to 9.04 mg^.^g^−1^ DW in long-term cultures. Murthy et al. (2018) reported the commercial production of *P. ginseng* adventitious roots in South Korea, which yield up to 32.46 mg^.^g^−1^ DW ginsenosides; according to the authors the annual production of root biomass amounts to 45 tons and is used in the pharmaceutical, food and cosmetic industry. The influence of various abiotic and biotic elicitors on ginsenoside formation in cell suspensions of *P. quinquefolius* was investigated by Biswas et al. (2016). The elicitor treatments were found to differentially impact total ginsenoside content, proportions of protopanaxadiols and protopanaxatriols, and the extent of product secretion into the culture medium. While the highest productivity (87.6 mg^.^L^−1^) was achieved upon a five-day treatment with a culture filtrate of *Trichoderma atroviridae*, a 15-day elicitation with *Trichoderma harzianum* led to maximum biosynthesis and secretion of ginsenoside Rh1, a compound with well-studied antioxidant, anti-inflammatory, immunomodulatory activities, and positive effects on the nervous system (Tam et al. 2018). In another ginseng species (*P*. *sikkimensis*) Biswas and co-authors (2018) studied the effects of elicitation with preparations of two bacterial and two fungal strains on ginsenoside biosynthetic genes and product formation. They achieved a yield of 222.2 mg^.^L^−1^ ginsenosides upon elicitation of cell suspensions with culture filtrate of the fungus *T*. *harzianum*. As mentioned above, ginseng is a highly popular plant in Asian countries. Studies on the in vitro production started in the 1960s and numerous papers have been published since. In terms of ginsenoside production, an excellent yield of ca. 1.5 g^.^L^−1^ with suspension cultures *of P. notoginseng* has already been reported more than 20 years ago (Zhang and Zhong 1997).

The solanaceous plant *Withania somnifera,* as well as other members of the family used medicinally in the Indian Ayurvedic medicinal system, contains up to 0.5% withanolides, biologically active steroidal lactone triterpenoids. The withanolide accumulation in various in vitro cultures has been investigated, and the formation of 14.2 mg^.^g^−1^ DW withanolides in cell suspension cultures of *W. somnifera* upon elicitation with a culture filtrate of *Piriformospora indica* has been reported by Ahlawat et al. (2017). In comparison, by combining elicitation with chitosan and precursor feeding (squalene) Sivanandhan et al. (2014) had achieved the production of 27.49 mg^.^g^−1^ DW withanolides. However, the withanolide content in the intact plant can vary considerably between 0.001 to 0.5% (Mirjalili et al. 2009), hence product formation rate in a cell suspension culture could also depend on the respective properties of the donor plant.

The pentacyclic triterpenoids, betulinic acid, oleanolic acid, and ursolic acid, are important raw materials for the preparation of semi-synthetic drugs, as has recently been reviewed for betulinic acid (Borkova et al. 2018). Upon elicitation with culture medium filtrate of *Aspergillus niger*, combined with sucrose fed-batch, Kümmritz et al. (2016) obtained 5.76 and 10.77 mg^.^g^−1^ DW oleanolic acid and ursolic acid, respectively, in suspension cultures of *Salvia fruticosa*. This was about 1/2 and 1/3 of the content found in the intact plants (Kümmritz et al. 2014). *Lantana camara* L. (*Verbenaceae*) is a popular ornamental plant which, however, is also known as a notorious weed. It has been used since a long time as medicinal plant in various parts of the world (Ghisalberti 2000). Kumar et al. (2016) investigated the accumulation of triterpenoids in suspension cultures of *L. camara* after elicitation with the root endophytic fungus *Piriformospora indica*. As stated by the authors, this was part of a study of the elicitor potential of the fungus. Treatment with a filter-sterilized culture filtrate of the fungus resulted in the formation of 117.02 µg^.^g^−1^ DW betulinic acid, 1.4 mg^.^g^−1^ DW oleanolic acid, and 3.87 mg^.^g^−1^ DW ursolic acid. Basically, the suitability of betulinic acid as a target compound for in vitro production has to be critically assessed. An efficient strategy for the preparation of the compound from plant raw material has recently been communicated by Ressmann et al. (2017). The precursor triterpenoid betulin is found in the bark of white birch in quantities up to 35%. Huge amounts of birch bark accumulate as by-product from pulp and paper mills, and while the conventional conversion of betulin to betulinic acid is problematic (low yield, toxic reagents), Ressmann et al. (2017) developed a quick extraction procedure for betulin at room temperature, followed by direct oxidation of the crude extract without using toxic reagents.

The flower heads of marigold (*Calendula officinalis*, *Asteraceae*) are used for the treatment of skin irritations and to promote wound healing. Besides other metabolites they also contain up to 10% triterpene saponins (Lichius et al. 2016). The accumulation of oleanolic acid glycosides in hairy roots of marigold was studied by Alsoufi et al. (2019). Elicitation with 100 µM jasmonic acid resulted in dramatic increase in productivity.

The medicinal use of *Ginkgo biloba* has been described ca. 5000 years ago in the Chinese pharmacopoeia Chen Noung Pen Tsao, and nowadays its efficacy in the treatment of Alzheimer’s disease, vascular dementia and other ailments is established (Volk et al. 2016). In a recent study Sukito and Tachibana (2016) investigated the productivity of ginkgo cells immobilized on jute fibers, combined with methyl jasmonate and salicylic acid elicitation. Production of 78 mg^.^g^−1^ DW bilobalide, 79 mg^.^g^−1^ DW ginkgolide A, 71 mg^.^g^−1^ DW ginkgolide B, and 7.5 mg^.^g^−1^ DW ginkgolide C could be achieved.

The Asteraceae *Stevia rebaudiana* is a well-known herb with an intensely sweet taste which is used to prepare sweeteners for home and commercial purposes. The leaves contain up to 10% of the diterpene glycoside, stevioside, as well as up to 4% rebaudioside A, 2% rebaudioside C, and 0.7% dulcoside as sweet tasting compounds (Blaschek and Loew 2016). Ahmad et al. (2018) investigated the influence of pH levels on product formation in adventitious root cultures. They found that lower pH (5.1) promoted the formation of stevioside (79.48 mg^.^g^−1^ DW) and rebaudioside A (13.10 mg^.^g^−1^ DW), while dulcoside could not be detected. At higher pH of 5.8 the concentration of the two former metabolites was significantly lower, but 2.57 mg^.^g^−1^ DW dulcoside were found. However, the pH level also influenced root growth, at pH 6.0 the highest biomass was observed. It is however questionable whether a production of steviosides through plant tissue culture would ever be competitive to the extraction from the plant, given the high content of up to 10% in the leaves.

The perennial herb *Hybanthus enneaspermus* is used in the Ayurveda medicinal system for the treatment of various disorders, like, e.g., urinary infections, dysentery, or cough (Patel et al. 2013). One of the secondary metabolites found in this plant is L-Dopa (L-3,4-dihyroxyphenylalanine) which is an important drug for the treatment of Parkinson’s disease. Sathish et al. (2020) investigated the effects of elicitors on the accumulation of L-Dopa in adventitious roots of *H. enneaspermus*. A treatment of the cultures with 100 µM salicylic acid resulted in the formation of 12.64 mg^.^g^−1^ DW L-Dopa. The authors suggested that adventitious root cultures of *H. enneaspermus* could be used for the industrial production of L-Dopa, it is however disputable whether this would be an alternative to microbial fermentation which exhibits considerably higher productivity (Min et al. 2015).

While the low productivity of cell suspension cultures remains a challenge, these recent studies show that upon proper optimization of suitable cell lines it is possible to achieve levels of secondary metabolites equal to or even higher than those found in the intact plant. Hence, considering the various advantages of biotechnological production of natural products, ongoing research in the field of plant cell suspension cultures remains an important task.

## Plant metabolic engineering

Plant metabolic engineering as relevant to bioactive secondary metabolites aims at increasing the accumulation of a desired product, or/and at reducing accumulation of an undesired metabolite. This can be achieved by overexpression of enzymes, either in upstream pathways, or in the target compound pathway; by increasing the activity of rate-limiting enzymes; by suppression of competing pathways or catabolic steps; and by creating sink compartments that store the desired metabolite (Farré et al. 2014). The modification of the tropane alkaloid pattern in *Atropa belladonna* was the first proof of concept for the successful metabolic engineering of medicinal plants. Through overexpression of *h6h*, the gene coding for hyoscyamine 6-ß hydroxylase which catalyzes the final steps in the biosynthesis of scopolamine, Yun et al. (1992) obtained *A. belladonna* plants with significantly elevated scopolamine content. Some of the most recent studies dealing with metabolically engineered plant in vitro cultures are summarized below.

Several studies with alkaloid-containing *Solanaceae* plants have shown that undifferentiated plant in vitro cultures like callus and cell suspensions do not accumulate significant amounts of tropane alkaloids (Kohnen-Johannsen and Kayser 2019). This is, however, not the case with hairy roots, and the transformation process with *A*. *rhizogenes* can also be utilized for metabolic engineering purposes. Species of the genus *Duboisia* are cultivated for the extraction of scopolamine, but besides tropane alkaloids, the roots of *Duboisia* species also synthesize nicotine and related pyridine alkaloids. Singh et al. (2018) demonstrated that RNAi silencing of the quinolinate phosphoribosyl transferase gene in the nicotine biosynthetic pathway led to significant inhibition of nicotine biosynthesis and enhanced formation of scopolamine. Upon elicitation of such a hairy root line with methyl jasmonate the production of 19.34 mg^.^g^−1^ DW scopolamine was achieved, compared to 1.44 mg^.^g^−1^ DW in the non-elicited wild type control line. In order to enhance the formation of the anticancer alkaloid camptothecin in hairy roots of *Ophiorrhiza pumila*, Shi et al. (2020) established a transgenic root line overexpressing geraniol-10-hydroxylase and secologanin synthetase genes. While under optimized culture conditions camptothecin production in stem and roots of up to 2 mg^.^g^−1^ DW has been reported (Lee et al. 2020), the engineered hairy root line yielded 3.5 mg^.^g^−1^ of the compound.

Biosynthesis of the pharmaceutically important terpenoid indole alkaloids (TIAs) in Madagascar periwinkle (*C*. *roseus*) involves a pathway of more than 35 enzymatic steps in different cellular and subcellular compartments (Sharma et al. 2020). Saiman et al. (2018) overexpressed the *C. roseus* geraniol synthase gene in either plastids or cytosol of a non-alkaloid producing *C. roseus* cell line. These cell lines produced TIAs upon elicitation with jasmonic acid, which indicated that other, inducible genes need to be expressed, too. The authors also found that alterations in the primary metabolism occurred, so that further studies are necessary to understand the full effects of their metabolic engineering approach. Sharma et al. (2018) introduced additional copies of the tryptophan decarboxylase and strictosidine synthase genes into *C. roseus* callus and achieved a doubled total alkaloid content of 1.2 mg^.^g^−1^ DW, when compared to the untransformed control. Sun and Peebles (2016) established *C. roseus* hairy roots overexpressing the transcriptional regulator ORCA3 (octadecanoid responsive *Catharanthus* AP2-domain protein) gene and the strictosidine glucosidase gene, which resulted in 47% increase in the content of six root-specific TIAs. In a different approach, overexpression of the CrTPT2 (a ATP-binding cassette transporter for catharanthine) gene in *C. roseus* hairy roots increased the accumulation of catharanthine to ca. 5 mg^.^g^−1^ DW, which was fivefold higher than in the wild type control roots (Wang et al. 2019).

An efficient plant cell-based production system for anthocyanins has been reported by Appelhagen et al. (2018). They established suspension cultures from a transgenic line of *Nicotiana tabacum* which constitutively expressed the *Am*Ros1 and *Am*Del transcription factors from *Antirrhinum majus* (Kallam et al. 2017). These cells produced 30 mg^.^g^−1^ DW cyanidin 3-O-rutinoside and this biosynthetic potential remained stable over a 10-years observation period. Upon additional introduction of a gene encoding flavonoid 3′,5′-hydroxylase from *Petunia* x *hybrida*, the resulting cell suspension culture was shown to produce cyanidin 3-O-rutinoside and also delphinidin 3-O-rutinoside. Engineering of a gene encoding an anthocyanin 3-O-rutinoside-4′′′-hydroxycinnamoyl transferase from *Solanum lycopersicum* into the *Am*Ros1/*Am*Del tobacco line led to the formation of acylated anthocyanins. The authors thereby demonstrated the potential of their system for the production of a variety of highly-decorated anthocyanins.

The abovementioned studies stress the importance of understanding the biosynthetic pathways for plant secondary metabolites in order to rationally manipulate them. In addition, understanding of pathway regulation, which can be very complex, is also required for optimal metabolic engineering of plants toward enhanced production of medicinally important compounds. For more detailed information, the reader is referred to relevant literature like, e.g., the recent review by Birchfield and McIntosh (2020).

## Heterologous production in microorganisms

Notwithstanding the fact that high yielding cell suspension and organ lines can be established for a variety of plants, plant tissue cultures suffer from a number of disadvantages that limit the large-scale commercial application to but a few processes. Cell suspension cultures often exhibit slow growth and frequently are genetically unstable, which results in unreliable productivity. Organ cultures are even slower in growth, and the large-scale cultivation especially of (hairy) root cultures is technically challenging and expensive. In contrast, microbial systems feature rapid growth, are readily scalable to established fermentation and downstream processes, and protocols for their genetic manipulation are readily available. Purification of the product is easier, and the production process is cost-effective because inexpensive feedstock can be utilized for fermentation media (Moses et al. 2017). This section highlights recent examples of the heterologous production of bioactive plant secondary metabolites in microbial hosts.

The indole alkaloid psilocybin is a psychoactive secondary metabolite from hallucinogenic mushrooms with very promising therapeutic properties for the treatment of depression, post-traumatic stress disorder, or addiction. In 2017 four psilocybin biosynthetic enzymes were characterized and an enzymatic synthesis of this metabolite from 4‐hydroxy‐l‐tryptophan was reported (Fricke et al. 2017). Subsequently, Hoefgen et al. (2018) achieved psilocybin titers of up to 110 mg^.^L^−1^ through heterologous expression of the biosynthetic genes in *Aspergillus nidulans*. In the most recent study, a modular biosynthetic production platform in *Escherichia coli* was constructed, and after fermentation optimization a production titer of 1.16 g^.^L^−1^ psilocybin by bioconversion of the precursors 4-hydroxyindole, serine and methionine was reported by Adams et al. (2019). Complete de novo production of psilocybin and related tryptamine derivatives in *Saccharomyces cerevisiae* was achieved by Milne et al. (2020). After introduction of five heterologous genes (tryptophan decarboxylase from *Catharanthus roseus* and four genes from *Psilocybe cubensis*) into yeast, followed by further engineering of genes in the shikimate pathway and optimization of fed-batch fermentation, the yeast strain yielded 627 mg^.^L^−1^ psilocybin and 580 mg^.^L^−1^ psilocin, the latter being the bioactive metabolite of psilocybin which is formed in humans from orally administered psilocybin.

Noscapine is one of the main alkaloids of opium poppy (*Papaver somniferum*). It is used as antitussive drug and has been shown to possess promising anticancer properties (DeBono et al. 2015). An engineered strain of *S. cerevisiae* was constructed containing 25 heterologous plant, mammalian and bacterial genes, and 6 overexpressed or mutant yeast genes. Through a combination of enzyme engineering, pathway and strain engineering, and fermentation optimization, a noscapine titer of 2.21 mg^.^L^−1^ could be achieved (Li et al. 2018). Notably, halogenated benzylisoquinolide alkaloids could be obtained by feeding modified tyrosine derivatives, which may lead to novel structures for drug development.

Kallscheuer et al. (2016) described the construction of a *Corynebacterium glutamicum* strain for the production of plant polyphenols. After deletion of genes involved in the catabolism of aromatic compounds, and expression of plant-derived genes coding for a chalcone synthase and a chalcone isomerase, they achieved the production of 35 mg^.^L^−1^ naringenin and 37 mg^.^L^−1^ eriodictyol from supplemented phenylpropanoids p-coumaric acid and caffeic acid, respectively. Upon further engineering of the amino acid metabolism they obtained strains which produced of 32 mg^.^L^−1^ naringenin or 59 mg^.^L^−1^ resveratrol, directly from glucose. In a further development of these strains, introduction of plant-derived dioxigenase genes into the flavanone producing strain enabled the production of 23 mg^.^L^−1^ kaempferol and 10 mg^.^L^−1^ quercetin. Similarly, by expression of a modified O-methyltransferase from grapevine in the previously engineered resveratrol producer, Kallscheuer and co-authors (Kallscheuer et al. 2017) achieved a titer of 42 mg^.^L^−1^ of the di-O-methylated compound pterostilbene.

Li et al. (2019) developed co-cultures of multiple engineered *E. coli* strains (modular co-culture engineering) for the production of rosmarinic acid. They divided the rosmarinic acid biosynthesis in three modules, namely caffeic acid, salvianic acid A, and rosmarinic acid module, which were engineered into separate *E. coli* strains. After optimization of strain-to-strain ratios and culture conditions, the production of 172 mg^.^L^−1^ rosmarinic acid was achieved, which was a 38-fold improvement over the parent strain used for monoculture-based biosynthesis. Acetylsalicylic acid (aspirin), a most prominent non-steroidal anti-inflammatory drug, is produced industrially through acetylation of salicylic acid. Starting from an engineered salicylic acid producing *E. coli* strain, Ahmadi et al. (2016) further introduced a codon-optimized glucosyltransferase gene from *Arabidopsis thaliana*. By a co-culture of two engineered strains the authors achieved a titer of ca. 2.5 g^.^L^−1^ salicylate 2-O-β-D-glucoside, which showed nitric oxide and reactive oxygen species reducing activities comparable to aspirin.

Hemp (*Cannabis sativa* L.) is one of the oldest crop plants on earth and has been used medicinally before being classified as illegal psychoactive drug. However, the therapeutic potential of some of its secondary metabolites, especially Δ9-tetrahydrocannabinol and cannabidiol, has been recognized and drugs for the treatment of multiple sclerosis symptoms as well as some forms of pediatric epilepsy have been approved (Cristino et al. 2020). In the cannabinoid biosynthesis, geranyl diphosphate and olivetolic acid are initial precursors which are condensed to cannabigerolic acid (CBGA). This intermediate is further converted to cannabidiolic acid (CBDA), Δ9-tetrahydrocannabinolic acid (Δ9-THCA) and cannabichromenic acid (CBCA) (Flores-Sanchez and Verpoorte 2008). In their attempts to establish a platform for the heterologous production of cannabinoids in yeasts, Zirpel et al. (2015) initially showed that *Pichia pastoris* expressing Δ9-THCA synthase from *C. sativa* produced Δ9-THCA from CBGA in a whole cell bioconversion assay. Subsequently, this yeast strain was further engineered with the prenyltransferase NphB from *Streptomyces* sp. strain CL190 and was shown to produce Δ9-THCA when supplemented with olivetolic acid and geranyl diphosphate (Zirpel et al. 2017). The complete biosynthesis of cannabinoids, and also of unnatural cannabinoid analogues, in engineered *S. cerevisiae* has been recently reported by Luo et al. (2019). This was accomplished by introducing a heterologous hexanoyl-CoA biosynthetic pathway, and providing a high flux of geranyl diphosphate by engineering the native mevalonate pathway. Furthermore, *Cannabis* genes for the biosynthesis of olivetolic acid, as well as genes for cannabinoid synthases were introduced. With an optimized strain, titers of 8 mg^.^L^−1^ Δ9-THCA were obtained. The authors also demonstrated that different fatty acid precursors were incorporated into corresponding olivetolic acid, CBGA and Δ9-THCA analogues. Such unnatural cannabinoids are of great interest because of potentially improved medicinal properties (Luo et al. 2019).

Heterologous production of the antimalarial drug artemisinin in microbial hosts has been intensely investigated and in 2013 led to the commercialization of “semi-synthetic artemisinin”: the precursor artemisinic acid was produced with an engineered strain of *S*. *cerevisiae* at 25 g^.^L^−1^ and then chemically converted to artemisinin (Kung et al. 2018). However, the process was not viable because of the then lower price of plant-derived drug in the fluctuating artemisinin market (Peplow 2016). In their search for alternative microbial hosts for artemisinin production, Marsafari and Xu (2020) chose the yeast *Yarrowia lipolytica* which they engineered to accumulate amorphadiene, which is the direct sesquiterpene precursor of artemisinin. Upon introduction of the codon-optimized *A. annua* amorphadiene synthase gene, the initially low amorphadiene titer could be increased to 171.5 mg^.^L^−1^ by introducing additional copies of some mevalonate pathway genes and inhibiting fatty acids synthesis.

Several recent studies dealt with the heterologous production of plant triterpenoids. Wang et al. (2020) constructed a synthetic biology platform to elucidate the saponin biosynthesis pathway of *Panax notoginseng.* Subsequently, they engineered a yeast cell factory that yielded 1.17 g^.^L^−1^ of ginsenoside compound K, an anticarcinogenic and anti-inflammatory secondary metabolite, the content of which is quite low in ginseng. Plants of the *Cucurbitaceae* family are widely used in Asian traditional medicine to treat various types of cancers, as well as inflammatory diseases. They contain cucurbitane-type triterpenoid glycosides derived from cucurbitadienol, which itself also has anticancer and anti-inflammatory properties (Li et al. 2017). A synthetic pathway for cucurbitadienol biosynthesis was constructed in *S. cerevisiae* by Qiao et al. (2019). Firstly, they introduced the cucurbitadienol synthase gene from the *Cucurbitaceae* plant *Siraitia grosvenorii* into a yeast strain with high squalene content. Subsequently, several genes involved in squalene biosynthesis were overexpressed, as squalene is a precursor in triterpene biosynthesis. Finally, the metabolic flux to ergosterol was downregulated through knockout of the ERG7 gene, thus resulting in the production of 63 mg^.^L^−1^ cucurbitadienol in fed-batch fermentation of the optimized yeast strain. Enhanced heterologous production of another triterpenoid, oleanolic acid, has been accomplished in yeast by Zhao et al. (2018). Three plant genes, namely a β-amyrin synthase gene from *Glycyrrhiza glabra*, an oleanolic acid synthase gene from *Medicago truncatula*, and a cytochrome P450 reductase gene from *M. truncatula*, were expressed in *S. cerevisiae* JDY52, thus resulting in the synthesis of oleanolic acid. Subsequently, overexpression of three genes of the yeast native ergosterol pathway, knockout of two genes involved in the galactose regulatory network, and optimization of the fermentation conditions resulted in a titer of 606.9 mg^.^L^−1^ oleanolic acid.

## Conclusions

For the industrial production of plant secondary metabolites, plant cell and organ culture provides a controllable, environmentally friendly, and sustainable alternative to laborious field cultivation. The large number of studies on the in vitro production of bioactive plant compounds published in the past few years indicates the unabated importance of relevant research. Factors like culture techniques or impact of elicitation on gene expression appear to be of outmost importance, and for a number of medicinal plants cell or organ cultures producing higher amounts of relevant metabolites than the respective plant have been reported. While many authors emphasize the suitability of their in vitro systems for the commercial production of the respective compound, such claims should be considered with care. Even after decades of research resulting in many high yielding cell suspension and organ culture lines, to date only one process, namely the production of paclitaxel by *Taxus* cell cultures, is deployed on an industrial scale. Quite a number of the recently published studies deal with secondary compounds which have been the subject of intense investigations since roughly 50 years, and highly productive in vitro systems have been published decades ago. Yet, although these processes were clearly superior to recently published systems, they have never been commercialized. In this view, it has to be realized that in most cases progress towards increased productivity of plant based in vitro systems is lacking.

A number of critical factors which determine the commercialization of plant in vitro production systems have to be considered, such as market prices (both of the pure compound, and of the raw material used for extraction of the compound), economical feasibility of chemical synthesis, regulatory requirements, and consumer acceptance. Both the market situation and the clinical demand for certain plant secondary metabolites are prone to changes (Lange 2018; Kreis 2019), and the extent of ongoing research as reviewed here demonstrates the potential of plant in vitro cultures for the production of bioactive secondary metabolites. Without a doubt, the metabolic engineering of plants and heterologous production in microorganisms are very promising approaches, that heavily depend on the elucidation of biosynthetic pathways, synthetic biology tools and progress in genome engineering. Especially the construction of microbial cell factories can be expected to render a cost-efficient production of bioactive plant secondary metabolites possible.

## Data Availability

Not applicable.
